# Bazedoxifene Attenuates Isoproterenol-Induced Cardiac Fibroblast Activation and Fibrotic Phenotype via Modulation of the IL-6/STAT3 Signaling Axis

**DOI:** 10.3390/cimb48090879

**Published:** 2026-08-29

**Authors:** Xiangyun Chen, Taomei Yang, Xiaofang Tang, Mengyue Guo, Lixue He, Yaofeng Li

**Affiliations:** 1 School of Basic Medicine, Guizhou University of Traditional Chinese Medicine, Guiyang 550025, China; chenxiangyun017@gzy.edu.cn (X.C.); yangtaomei017@gzy.edu.cn (T.Y.); tangxiaofang046@gzy.edu.cn (X.T.); 18685527239@163.com (L.H.); 2 School of Traditional Chinese Medicine, Guizhou University of Traditional Chinese Medicine, Guiyang 550025, China; 19912820157@163.com

**Keywords:** bazedoxifene, cardiac fibroblasts, IL-6/STAT3 signaling, myocardial fibrosis, isoproterenol, drug repurposing

## Abstract

Bazedoxifene (BAZ), a selective estrogen receptor modulator, has recently been demonstrated to inhibit the IL-6/STAT3 signaling pathway; however, its direct effects on cardiac fibroblasts and the underlying mechanisms remain unclear. Isoproterenol (ISO)-stimulated Sprague–Dawley neonatal rat cardiac fibroblasts (CFs) were employed as an in vitro model. CCK-8 assay, flow cytometry, Transwell migration assay, ELISA, qRT-PCR, and Western blot were applied to evaluate the effects of BAZ on CF activation, proliferation, migration, and collagen synthesis. Additionally, IL-6 overexpression via lentivirus (Lv-IL-6) was used to assess mediation by IL-6/STAT3 signaling. BAZ (5 μmol/L) significantly inhibited ISO-induced CF proliferation by inducing G_0_/G_1_ cell-cycle arrest; migration and upregulation of α-SMA and Collagen I/III were reduced; IL-6, TGF-β1, and hydroxyproline concentrations in the conditioned medium were decreased; STAT3 phosphorylation was significantly suppressed. Supplementation with Lv-IL-6 partially reversed these effects. The suppression of ISO-induced CF activation and fibrotic phenotype was associated with inhibition of IL-6 expression and blockade of IL-6/STAT3 signaling, suggesting the involvement of this pathway in the anti-fibrotic effects of BAZ. These findings provide in vitro evidence supporting BAZ as a candidate anti-myocardial fibrosis agent.

## 1. Introduction

Myocardial fibrosis represents a common pathological process underlying the progression of diverse cardiovascular diseases toward heart failure [1]. It is characterized by the aberrant activation, proliferation, and differentiation of cardiac fibroblasts (CFs) into myofibroblasts. These myofibroblasts secrete substantial amounts of extracellular matrix (ECM) proteins, leading to excessive interstitial collagen deposition, increased ventricular wall stiffness, and subsequent impairment of both systolic and diastolic function [2]. Myocardial fibrosis is observed in hypertensive heart disease, myocardial infarction, heart failure, and hereditary cardiomyopathies, and it serves as an important predictor of arrhythmia and sudden cardiac death. Despite progress in basic and clinical research, effective therapeutic interventions specifically targeting myocardial fibrosis remain unavailable [3]. During the progression of fibrosis, cardiac fibroblasts act as the principal effector cells. Upon pathological stimulation, quiescent CFs become activated and transdifferentiate into myofibroblasts expressing α-smooth muscle actin (α-SMA), thereby acquiring proliferative, migratory, and collagen-secreting capabilities [4]. Elucidating the molecular regulatory mechanisms that govern CF activation and identifying pharmacological strategies directed at CFs hold significant scientific and translational value for the prevention and treatment of myocardial fibrosis.

Interleukin-6 (IL-6), a pleiotropic proinflammatory cytokine, has been established as a pivotal regulator of myocardial fibrosis [5]. Significant upregulation of IL-6 is observed in cardiac tissue and CFs derived from both myocardial infarction (MI) patients and murine models [6]. Selective ablation of IL-6 in fibroblasts markedly attenuates MI-induced cardiac fibrosis, adverse ventricular remodeling, and preserves cardiac function [7]. Mechanistically, the binding of IL-6 to IL-6Rα and its co-receptor gp130 activates downstream JAK/STAT3 signaling [8]; phosphorylation of STAT3 facilitates its nuclear translocation and subsequent transcriptional regulation of profibrotic target genes [9]. Evidence suggests that IL-6 exacerbates post-MI cardiac fibrosis, at least partially, through activation of a JAK1/STAT3 signaling axis [10,11]. Additionally, macrophage-mediated stimulation of CFs promotes IL-6 production, which in turn enhances transforming growth factor-beta 1 (TGF-β1) synthesis and Smad3 phosphorylation, establishing a profibrotic positive-feedback loop [12,13]. Under conditions such as chronic β-adrenergic stimulation and other pathological states, aberrant activation of IL-6/STAT3 signaling similarly drives myocardial fibrosis [11]. Collectively, these findings highlight IL-6/STAT3 signaling as a critical target for the development of anti-fibrotic therapeutic agents.

Bazedoxifene (BAZ), a third-generation selective estrogen receptor modulator (SERM), is approved in the United States only as a fixed-dose combination with conjugated estrogens (DUAVEE^®^) for the prevention of postmenopausal osteoporosis [14]. Notably, recent studies have revealed pharmacological activities beyond its SERM function, including direct interaction with the gp130 receptor and inhibition of IL-6- and IL-11-mediated STAT3 signaling [8,15]. In oncology, BAZ has been characterized as an IL-6/GP130/STAT3 pathway inhibitor with broad antitumor potential [14]. In the cardiovascular system, Shi et al. reported that oral administration of BAZ (5 mg/kg) suppressed mRNA expression of fibrosis-associated genes (IL-6, Col1A1, Col3A1, MMP2) and inhibited STAT3 phosphorylation in pressure-overload murine hearts, thereby ameliorating myocardial remodeling and function [16]. In H9c2 cardiomyoblasts, BAZ inhibited IL-6-induced STAT3 activation [16]. Additionally, BAZ reversed isoproterenol (ISO)-induced cardiomyocyte STAT3-Y705 phosphorylation, reactive oxygen species generation, and apoptosis. In a chronic ISO-induced β-adrenergic stimulation murine model, BAZ (5 mg/kg/day) attenuated cardiac fibrosis to an extent comparable with carvedilol [11]. These findings indicate that BAZ may represent a promising candidate for anti-cardiac-fibrosis therapy.

However, the direct effects of BAZ on cardiac fibroblasts (CFs) and its underlying molecular mechanisms have not been systematically investigated. It remains unknown whether BAZ can directly inhibit ISO-induced CF activation, proliferation, and migration. Whether BAZ blocks aberrant IL-6/STAT3 pathway activation through downregulation of IL-6 expression in CFs has yet to be determined. Moreover, whether IL-6 mediates BAZ anti-fibrotic effects and can be validated by IL-6 overexpression complementation requires further investigation. Resolution of these questions would facilitate elucidation of the cellular and molecular mechanisms underlying BAZ-mediated anti-cardiac-fibrosis effects and provide an experimental basis for drug repurposing.

An in vitro model utilizing cardiac fibroblasts isolated from neonatal Sprague–Dawley (SD) rats was established, wherein isoproterenol (ISO) was employed to trigger fibroblast activation and inflammatory responses. A comprehensive investigation was carried out to assess the effects of BAZ on cardiac fibroblast proliferation, migration, activation, and collagen synthesis, with a particular focus on determining whether its anti-fibrotic properties are achieved through the suppression of IL-6 expression and subsequent inhibition of the IL-6/STAT3 signaling pathway. This study was designed to furnish experimental evidence supporting BAZ as a novel anti-myocardial-fibrosis agent that targets the IL-6/STAT3 axis, thereby broadening the pharmacotherapeutic strategies available for myocardial fibrosis. Notably, our study is the first to systematically investigate the direct effects of BAZ on cardiac fibroblasts and to provide causal evidence—through IL-6 overexpression rescue experiments—that the anti-fibrotic effects of BAZ are mediated via suppression of IL-6 expression.

## 2. Materials and Methods

### 2.1. Reagents

Rat Cardiac Fibroblasts (CP-R074) and Rat Cardiac Fibroblast Complete Medium (CM-R074) were obtained from Wuhan Procell Life Science & Technology Co., Ltd. (Wuhan, China). Isoprenaline hydrochloride (HY-B0468), Bazedoxifene (HY-A0031), and Propranolol hydrochloride (HY-B0573) were sourced from MedChemExpress Co., Ltd. (Monmouth Junction, NJ, USA). The Hydroxyproline (Hyp) Assay Kit (A030-1-1) was procured from Nanjing Jiancheng Bioengineering Institute (Nanjing, China). The Cell Counting Kit-8 (C0038), DAPI Staining Solution (C1006), Crystal Violet (Y268091), Cy3-labeled Goat Anti-Rabbit IgG (H+L) (A0516), Rat IL-6 Enzyme-Linked Immunosorbent Assay (ELISA) Kit (PI328), Rat TGF-β1 ELISA Kit (PT878), and BeyoFast™ SYBR Green qPCR Mix (D7265) were purchased from Shanghai Beyotime Biotechnology Co., Ltd. (Shanghai, China). Collagen I (139 kDa) Antibody (ab260043) and Collagen III (139 kDa) Antibody (ab184993) were acquired from Abcam Shanghai Trading Co., Ltd. (Shanghai, China); α-SMA (42 kDa) Antibody (BF9212), IL6 (24 kDa) Antibody (DF6087), Phospho-STAT3 (Tyr705, 86 kDa) Antibody (AF3293), STAT3 (86 kDa) Antibody (AF6294), and β-actin (42 kDa) Antibody (AF7018) were obtained from Affinity Biosciences (Changzhou, China).

### 2.2. Cell Culture and Identification

Cardiac fibroblasts were obtained from Wuhan Procell Life Science & Technology Co., Ltd. (CP-R074). The cells were isolated from neonatal (2 day old) Sprague–Dawley rats of both sexes (donor number: 5 rats per lot, pooled). Upon receipt, cells were tested negative for mycoplasma and used at passages 2–5 for all experiments. Three independent cell lots (Lot No. 20240315, 20240420 and 20240610) were used for the experiments; data from all lots were combined after confirming no significant lot-to-lot variation.

Thawed cardiac fibroblasts obtained from neonatal Sprague–Dawley rats were seeded into culture flasks containing complete culture medium and maintained at 37 °C in a humidified atmosphere of 5% CO_2_. Upon reaching approximately 80% confluence, the cells were passaged for subsequent experimental procedures. To assess cell purity, cardiac fibroblasts were plated onto sterile glass coverslips placed in six-well plates and cultured until they reached 70% confluence. The culture medium was then removed, and the cells were rinsed three times with phosphate-buffered saline (PBS), followed by fixation with 4% paraformaldehyde at room temperature for 15 min. After additional PBS washes, the cells were permeabilized with 0.2% Triton X-100 at room temperature for 10 min and subsequently blocked with 5% bovine serum albumin (BSA) at room temperature for 30 min. A rabbit anti-vimentin primary antibody (diluted 1:200) was applied and incubated overnight at 4 °C. Following PBS washes, a Cy3-conjugated goat anti-rabbit IgG (H+L) secondary antibody (diluted 1:500) was applied and incubated in the dark at room temperature for 1 h. After another set of PBS washes, the nuclei were counterstained with DAPI for 5 min. Coverslips were then mounted onto slides, and images were captured using fluorescence microscopy.

### 2.3. Drug Concentration Screening

An appropriate amount of BAZ powder was dissolved in sterile dimethyl sulfoxide (DMSO) to prepare a 100 mM stock solution, which was then aliquoted and stored at −80 °C protected from light. On the day of the experiment, the stock solution was serially diluted in serum-free medium to obtain final concentrations ranging from 1 nM to 100 μM, with the final DMSO concentration maintained at ≤0.1% (*v*/*v*); an equal-volume DMSO vehicle control (Veh) was included. All dilution procedures were performed under sterile conditions, and working solutions were prepared immediately before use.

To determine a safe and effective concentration of BAZ, its effects on cardiac fibroblast (CF) viability were assessed using the CCK-8 assay. Cells were seeded in 96-well plates and, after attachment, treated with medium containing BAZ at 1 nM, 10 nM, 100 nM, 1 μM, 10 μM, 50 μM, and 100 μM; a normal control (CTL) and an equal-volume DMSO vehicle control (Veh) were included. After 24 h of treatment, CCK-8 solution was added to each well, followed by incubation and measurement of absorbance at 450 nm using a microplate reader; relative cell viability was calculated according to the formula. Relative cell viability (%) = [(A_treatment_ − A_blank)/(A_C_tl_ − A_blank)] × 100%, where A represents the absorbance at 450 nm.

To screen the optimal concentration of BAZ for suppression of the inflammatory response, interleukin-6 (IL-6) concentration in cell culture supernatants was measured by ELISA. Cells were allocated to the following groups: normal control (CTL), vehicle control (Veh), ISO stimulation without DMSO (ISO (w/o DMSO)), isoproterenol model (ISO model), BAZ at 1 nM, 10 nM, 100 nM, 1 μM, 5 μM, and 10 μM combined with ISO, and positive control propranolol (PRO, 8 μM) combined with ISO. After 1 h of pretreatment with the respective agents, ISO (10 μM) was added for 48 h to establish an in vitro cardiac fibroblast fibrosis model [17]. Culture supernatants were collected, IL-6 levels were measured according to the ELISA kit instructions, absorbance per well was recorded, and IL-6 concentration was calculated from a standard curve.

### 2.4. Experimental Grouping

Based on preliminary results and CCK-8 toxicity screening, the subsequent mechanistic experiments were divided into the following groups: (1) normal control (CTL): no treatment; (2) vehicle control (Veh): addition of an equal volume of DMSO; (3) ISO alone stimulation (ISO (w/o DMSO)): addition of 10 μM ISO (without DMSO); (4) ISO model (ISO model): addition of 10 μM ISO (with an equal volume of DMSO); (5) 5 μM BAZ + ISO: pretreatment with 5 μM BAZ for 1 h, followed by addition of 10 μM ISO; (6) 5 μM BAZ + Lv-NC + ISO: after transduction with negative-control lentivirus (Lv-NC) for 72 h, pretreatment with 5 μM BAZ for 1 h was performed prior to addition of 10 μM ISO; (7) BAZ + Lv-IL-6 + ISO: after transduction with IL-6-overexpressing lentivirus for 72 h, pretreatment with 5 μM BAZ for 1 h was performed prior to addition of 10 μM ISO. Cells in each group were seeded at a uniform density of 2.5 × 10^5^ cells/well in 6-well plates and allowed to attach overnight before treatment.

### 2.5. Lentiviral Transduction and IL-6 Overexpression

Lentiviral vectors encoding the rat IL-6 gene (Lv-IL-6, Gene ID: 24498, NCBI Reference Sequence: NM_012589) and a negative control lentivirus (Lv-NC) were packaged and prepared by General Biological (Anhui) Co., Ltd. (Chuzhou, China). Cardiac fibroblasts in the logarithmic growth phase were seeded into 6-well plates 24 h prior to transduction, with cell density adjusted to achieve 60% confluence at the time of transduction. The culture medium was replaced with fresh complete medium containing either Lv-IL-6 or Lv-NC viral particles along with polybrene (final concentration 5 μg/mL), and transduction was carried out at a multiplicity of infection (MOI) of 30. After incubation at 37 °C with 5% CO_2_ for 12 h, the medium was replaced with fresh complete medium for continued culture. At 72 h post-transduction, IL-6 mRNA and protein expression levels were assessed by Real-Time quantitative reverse transcription PCR (qRT-PCR) and ELISA to evaluate the efficiency of overexpression; subsequent pharmacological intervention experiments were initiated only after confirmation of effective overexpression.

### 2.6. Flow Cytometric Analysis of Cell Cycle

Following respective treatments, cells from each group were harvested via digestion with 0.25% trypsin-EDTA and washed twice with ice-cold PBS. For fixation, ice-cold 70% ethanol was added dropwise, and samples were incubated at 4 °C overnight. After fixation, cells were washed with PBS to remove residual ethanol and resuspended in PBS containing RNase A (50 μg/mL), then incubated at 37 °C for 30 min. Propidium iodide (PI) was added to a final concentration of 50 μg/mL, and staining was conducted at room temperature in the dark for 30 min. DNA content distribution was assessed using a BD FACSCalibur flow cytometer (BD Biosciences, San Jose, CA, USA) equipped with BD CellQuest Pro software (version 6.0; BD Biosciences, San Jose, CA, USA). For each sample, 20,000 events were acquired. Doublet discrimination was performed using pulse-width vs. pulse-area gating. Cell-cycle analysis was performed using ModFit LT software (version 5.0; Verity Software House, Topsham, ME, USA).

### 2.7. Determination of Hydroxyproline Content

Culture supernatants from each group were collected. Samples were then centrifuged at 1000× *g* for 10 min at 4 °C to remove cell debris, and the resulting supernatants were retained for subsequent assay. The hydroxyproline concentration in each sample was determined strictly following the kit manufacturer’s instructions.

### 2.8. ELISA

IL-6 concentrations were measured using the Rat IL-6 ELISA Kit (PI328, Beyotime; standard curve range: 250–8000 pg/mL). TGF-β1 concentrations were measured using the Rat TGF-β1 ELISA Kit (PT878, Beyotime; standard curve range: 62.5–2000 pg/mL). Culture supernatants from each group were collected. The samples were centrifuged at 1000× *g* for 10 min at 4 °C to remove cell debris, and the resulting supernatants were kept for analysis. Samples were assayed without dilution, as all readings fell within the standard curve range. Standard curves were generated using four-parameter logistic regression (R^2^ > 0.99 for all runs). Intra-assay and inter-assay coefficients of variation were both <10%, as determined by the manufacturer. Concentrations of IL-6 and TGF-β1 in each sample were quantified using their respective ELISA kits, in strict accordance with the manufacturer’s instructions.

### 2.9. Transwell Migration Assay

Transwell inserts with 8 μm pore size were placed in 24-well plates. The upper chamber was filled with serum-free medium, while the lower chamber contained complete medium supplemented with 10% fetal bovine serum (FBS) as a chemoattractant. Cardiac fibroblasts (CFs) from each experimental group were subjected to their respective treatments, then harvested, resuspended in serum-free medium, and adjusted to a concentration of 2.5 × 10^5^ cells/mL. A 100 μL aliquot of the cell suspension was seeded into the upper chamber. The plates were incubated at 37 °C in a humidified atmosphere of 5% CO_2_ for 12 h. BAZ (5 μM) and ISO (10 μM) were present in both the upper and lower chambers throughout the 12 h migration period. Following incubation, the inserts were removed, and non-migrated cells on the upper surface of the membrane were gently removed with a cotton swab. The migrated cells were fixed with 4% paraformaldehyde for 15 min and stained with 0.1% crystal violet for 20 min. After washing with phosphate-buffered saline (PBS), five randomly selected high-power fields (200× magnification) per membrane were captured using an inverted microscope. Field selection was performed using a systematic random sampling approach, and counting was performed blinded to group assignment by two independent investigators. The number of migrated cells was quantified and averaged across the five fields. The membrane was treated as the experimental unit for statistical analysis.

### 2.10. qRT-PCR

Total RNA was isolated from each cell group following their respective treatments using TRIzol reagent. For cDNA synthesis, 1 μg of total RNA served as a template with a reverse transcription kit. Subsequently, cDNA was used as a template for qPCR amplification using SYBR Green qPCR premix on a real-time PCR system. Primer sequences were as follows: *IL-6* forward: 5′- CGATGATGCACTGTCAGAAAAC -3′, reverse: 5′- ACTCCAGGTAGAAACGGAACTC -3′; *β-actin* forward: 5′- TGACGTTGACATCCGTAAAGACC -3′, reverse: 5′- GTGCTAGGAGCCAGGGCAGTAA -3′ [18]. Primer amplification efficiencies were validated using standard curves (E = 95–105% for all primers). Melt-curve analysis was performed for each run to confirm single-product amplification. No-template controls and no-reverse-transcription controls were included as negative controls. The thermocycling protocol comprised an initial denaturation at 95 °C for 2 min, followed by 40 cycles of denaturation at 95 °C for 15 s, annealing at 60 °C for 30 s, and extension at 72 °C for 30 s. Relative *IL-6* mRNA expression levels were determined using the 2^−ΔΔCt^ method and normalized to *β-actin* expression. β-Actin was selected as the reference gene based on its stable expression across experimental conditions, as confirmed by preliminary experiments.

### 2.11. Western Blotting

Cells from each group were washed twice with ice-cold PBS and then lysed on ice for 30 min using RIPA lysis buffer supplemented with protease and phosphatase inhibitors. The lysates were centrifuged at 12,000× *g* for 15 min at 4 °C, and the resulting supernatants were collected. Protein concentration was determined via the BCA assay. Equal amounts of protein (30 µg per well) were resolved by 10% SDS-PAGE and transferred onto polyvinylidene difluoride (PVDF) membranes. Membranes were blocked with 5% nonfat milk at room temperature for 1 h, followed by overnight incubation at 4 °C with primary antibodies against α-SMA (1:1000), Collagen I (1:1000), Collagen III (1:1000), IL-6 (1:1000), p-STAT3 (Tyr705, 1:1000), STAT3 (1:1000), and β-actin (1:5000). After three washes with TBST, membranes were incubated with horseradish peroxidase–conjugated goat anti-rabbit secondary antibody (1:5000) at room temperature for 1 h. Signals were detected using enhanced chemiluminescence (ECL), and band densities were quantified with ImageJ software (version 1.54p; National Institutes of Health, Bethesda, MD, USA). Relative protein expression levels were normalized to β-actin. Phosphorylated STAT3 expression was interpreted relative to total STAT3 expression (p-STAT3/STAT3 ratio).

### 2.12. Statistical Analysis

All experiments were performed using at least three independent biological replicates (*n* = 3–6 as indicated in each figure legend), each derived from an independently cultured batch of neonatal rat cardiac fibroblasts. Data are presented as mean ± standard deviation (mean ± SD). Statistical analyses and figure generation were performed using GraphPad Prism 9.0. Data normality was assessed using the Shapiro–Wilk test (α = 0.05), supplemented by Q–Q plots and skewness/kurtosis examination due to limited power with small samples (*n* = 3). Homogeneity of variances was checked by Levene’s test. For multiple-group comparisons, one-way ANOVA followed by Tukey’s test was used for normally distributed data; otherwise, the Kruskal–Wallis test with Dunn’s post hoc test was applied. A *p* value < 0.05 was considered indicative of statistical significance.

## 3. Results

### 3.1. Identification of Cardiac Fibroblasts from Neonatal SD Rats and Screening of Optimal BAZ Concentration

**Figure 1:**
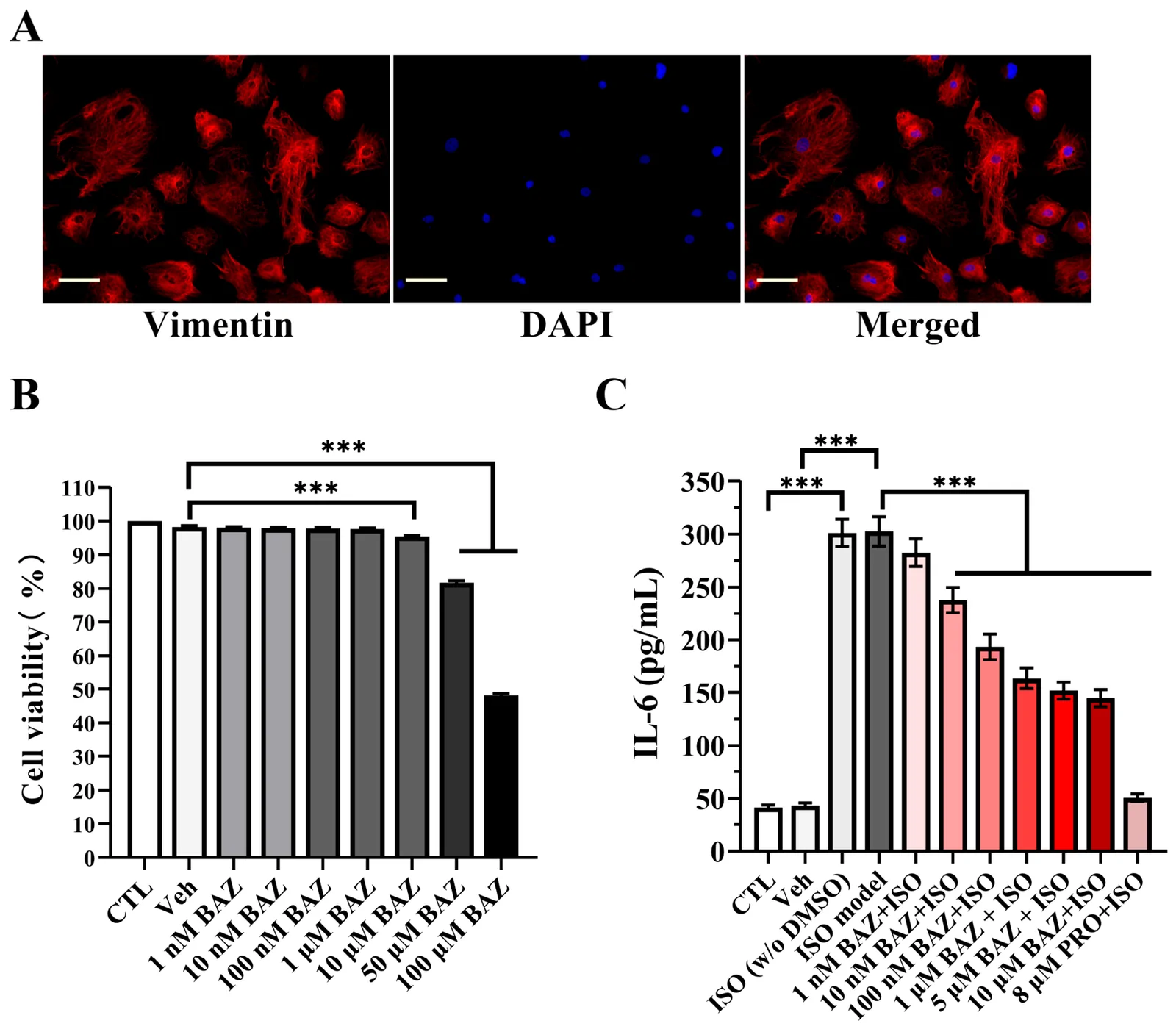
Identification of cardiac fibroblasts and determination of the optimal working concentration of bazedoxifene. (**A**) Representative immunofluorescence images of vimentin staining in neonatal Sprague–Dawley (SD) rat cardiac fibroblasts (CFs). Red fluorescence denotes vimentin-positive cells; blue DAPI staining labels nuclei. Scale bar = 100 μm. (**B**) Cell viability of CFs following 24 h exposure to bazedoxifene (BAZ, 1 nM–100 μM) or vehicle control (Veh, containing an equivalent volume of DMSO), evaluated using the CCK-8 assay. Data are expressed as mean ± SD (*n* = 6 independent experiments). (**C**) IL-6 secretion in culture supernatants of CFs was quantified by ELISA. Experimental groups comprised: CTL (normal control), Veh (DMSO vehicle), ISO (w/o DMSO) (ISO stimulation without DMSO), ISO model (ISO model with DMSO), 1–10 μM BAZ + ISO, and positive control PRO (8 μM propranolol + ISO). Data are presented as mean ± SD (*n* = 6 independent experiments). *** *p* < 0.001.

To ensure the reliability of subsequent experimental models, phenotypic validation was performed on cardiac fibroblasts purchased from neonatal SD rats. Vimentin immunofluorescence staining revealed that >98% of cells were vimentin-positive, with a distinct cytoplasmic filamentous signal (red) along with spindle-shaped or polygonal cell morphology, consistent with the cardiac fibroblast (CF) phenotype (Figure 1A). These results confirmed that cell purity met experimental requirements, allowing their use as an in vitro model for subsequent investigations.

Prior to evaluating drug effects, the safe concentration range of BAZ and potential solvent interference were determined. CCK-8 assay results demonstrated that, compared with the normal control (CTL), the vehicle control (Veh, containing an equivalent volume of DMSO) showed no statistically significant difference in cell viability. This confirmed the absence of DMSO cytotoxicity at the concentrations used and excluded solvent-related interference in subsequent pharmacological evaluations (Figure 1B). Under these conditions, low concentrations of BAZ (1 nM–1 μM) did not significantly affect cell viability; at 10 μM, viability decreased to 95.5% (*p* < 0.001); at 50 μM and 100 μM, viability declined markedly (*p* < 0.001). Accordingly, subsequent experiments were conducted using BAZ concentrations ≤ 10 μM, establishing this as a safe dosing range for investigating biological functions.

To determine the optimal concentration of BAZ for its anti-inflammatory effect, IL-6 levels were measured in the supernatants of ISO-induced CFs across a range of BAZ concentrations (Figure 1C). Basal IL-6 secretion in the CTL group was minimal, establishing a baseline under physiological conditions. In the ISO-alone stimulation group (without DMSO), the IL-6 concentration was significantly elevated compared to the CTL group (*p* < 0.001), confirming successful establishment of the ISO-induced inflammatory model and indicating that the proinflammatory effect was not driven by the solvent. The IL-6 level in the ISO model group (containing an equal volume of DMSO as used in BAZ treatments) did not differ significantly from the ISO-alone group, further excluding any modulatory effect of DMSO on ISO-induced inflammation and confirming that subsequent pharmacodynamic changes were attributable to BAZ. Based on these validation results, BAZ treatment exhibited a pronounced concentration-dependent inhibitory effect: as the concentration increased from 1 nM to 10 μM, IL-6 secretion progressively declined, with maximal inhibition observed at 10 μM (*p* < 0.001 vs. ISO model). No significant difference in inhibition was noted between 5 μM and 10 μM. Additionally, the positive control propranolol (PRO, 8 μM) elicited significant inhibition (*p* < 0.001 vs. ISO model). Considering both cellular safety (5 μM falls well within the safe range with minimal toxicity, as demonstrated in Figure 1B) and pharmacodynamic data (equivalent efficacy to 10 μM), 5 μM was designated as the optimal working concentration for subsequent experiments.

### 3.2. BAZ Inhibits ISO-Induced Cardiac Fibroblast Proliferation by Arresting the Cell Cycle

**Figure 2:**
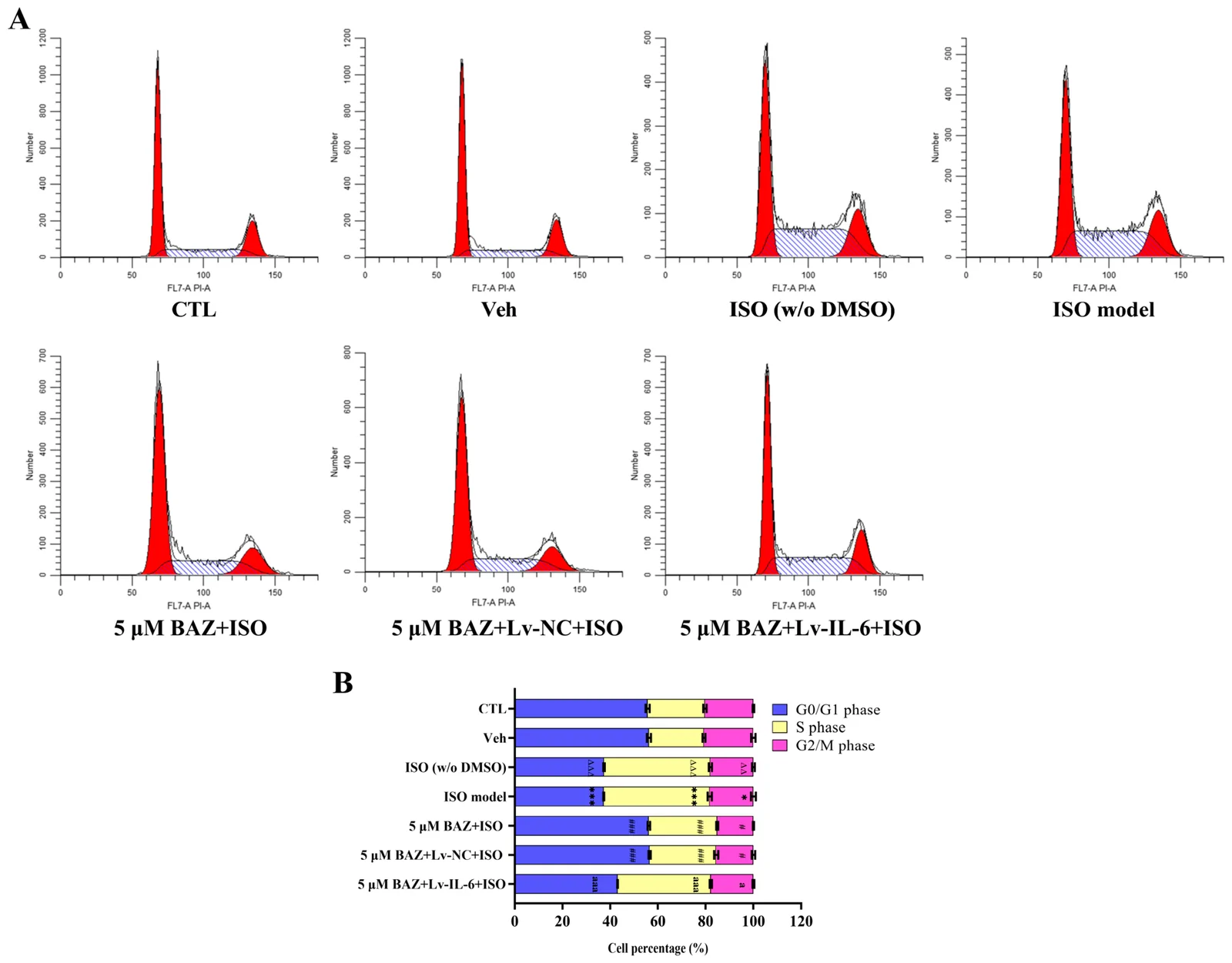
Bazedoxifene-mediated suppression of ISO-induced proliferation in cardiac fibroblasts was linked to G_1_-to-S phase cell cycle arrest, which was reversed by IL-6 overexpression. (**A**) Flow cytometric analysis was used to evaluate DNA content distribution across cardiac fibroblast (CF) groups. The experimental groups were defined as follows: CTL (normal control); Veh (equal-volume DMSO solvent control); ISO (w/o DMSO) and ISO model (10 μM ISO stimulation; the latter included an equal volume of DMSO to account for potential solvent effects); 5 μM BAZ + ISO; 5 μM BAZ + Lv-NC + ISO (negative lentiviral control); and 5 μM BAZ + Lv-IL-6 + ISO (IL-6 overexpression rescue). (**B**) Stacked bar chart illustrating the percentage of cells in each cell cycle phase (G_0_/G_1_, S, G_2_/M) for all groups. Data are expressed as mean ± SD (*n* = 3 independent experiments). ^ΔΔΔ^ *p* < 0.001, ^ΔΔ^ *p* < 0.01 vs. the CTL group; *** *p* < 0.001, * *p* < 0.05 vs. the Veh group; ^###^ *p* < 0.001, ^#^ *p* < 0.05 vs. the ISO model group; ^aaa^ *p* < 0.001, ^a^ *p* < 0.05 vs. the 5 μM BAZ + ISO group.

The aberrant activation and proliferation of cardiac fibroblasts are central events driving myocardial fibrosis progression. To investigate whether BAZ inhibits fibroblast activation and proliferation through cell cycle regulation, we analyzed cell-cycle distributions across experimental groups using flow cytometry (Figure 2). As illustrated in Figure 2A,B, compared with the CTL and Veh groups, the ISO-treated groups (ISO w/o DMSO and ISO model) displayed a significant reduction in the proportion of cells in the G0/G1 phase (*p* = 0.0002 or *p* = 0.0003) and a significant increase in the S-phase proportion (*p* = 0.00003 or *p* = 0.00001). These findings indicate that ISO promotes the transition of cardiac fibroblasts from quiescence into DNA synthesis and mitotic phases, thereby enhancing proliferative activity. The high concordance between the two ISO-treated groups confirms that the DMSO vehicle did not interfere with cell-cycle distribution. In comparison with the ISO model group, the 5 μM BAZ + ISO group exhibited a significant restoration of the G_0_/G_1_-phase proportion (*p* = 0.00007), accompanied by reductions in the S-phase and G_2_/M-phase proportions (*p* = 0.0002 or *p* = 0.0293), demonstrating that BAZ effectively inhibits ISO-induced G_1_-to-S phase transition in fibroblasts, thereby exerting antiproliferative effects. Furthermore, the cell-cycle distribution in the negative viral control group (5 μM BAZ + Lv-NC + ISO) did not significantly differ from that in the 5 μM BAZ + ISO group. To further determine whether BAZ’s antiproliferative effect depends on modulation of IL-6 signaling, we constructed an IL-6-overexpressing lentivirus (Lv-IL-6) for rescue experiments. Results revealed that, compared with the 5 μM BAZ + ISO group, the 5 μM BAZ + Lv-IL-6 + ISO group showed a significant decrease in the G_0_/G_1_-phase proportion (*p* = 0.0003) and significant increases in the S-phase and G_2_/M-phase proportions (*p* = 0.00001 or *p* = 0.0011), indicating that IL-6 upregulation partially reverses BAZ-mediated antiproliferative effects.

### 3.3. BAZ Significantly Downregulated ISO-Induced Hyp Production in Cardiac Fibroblasts

**Figure 3:**
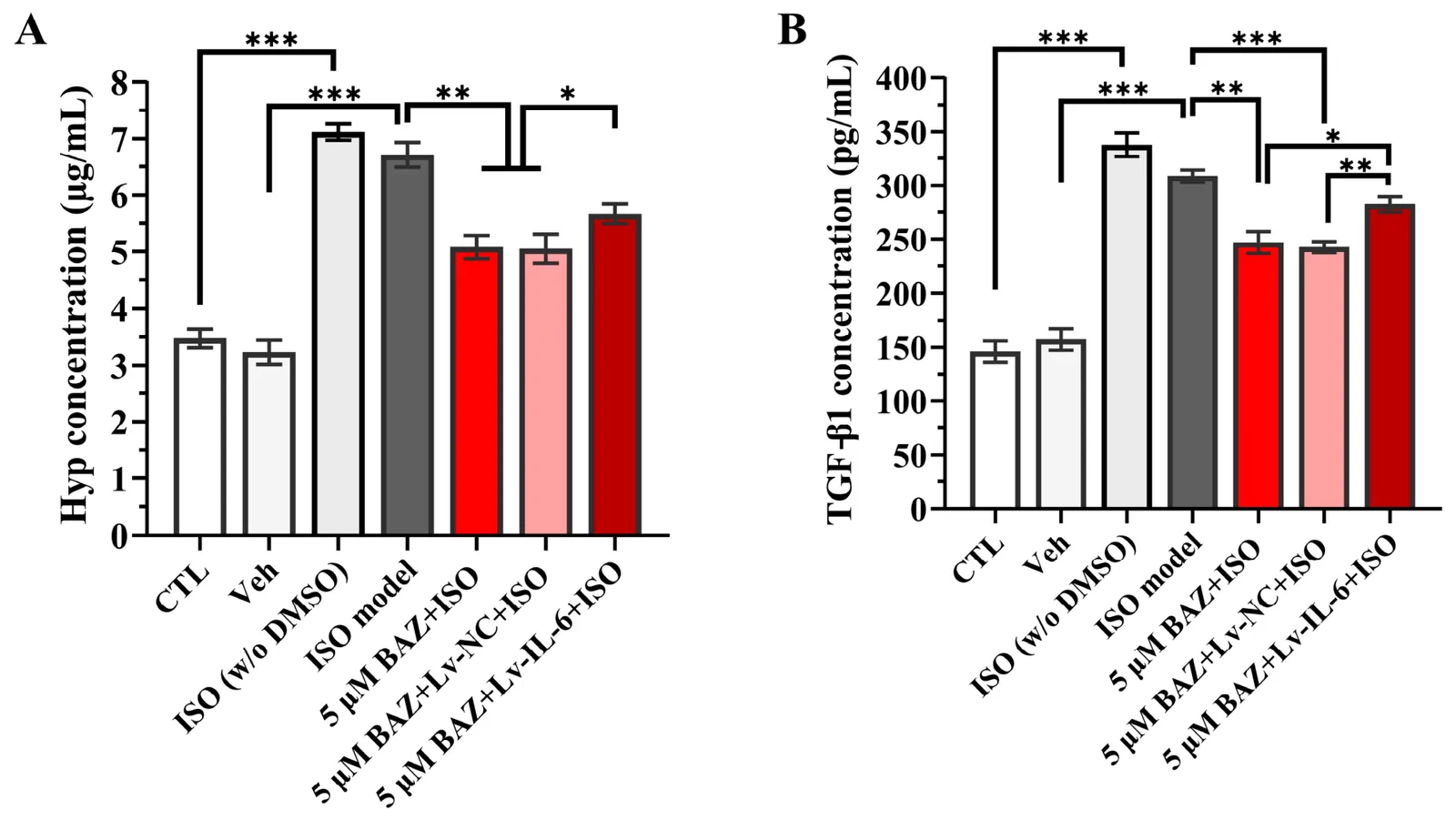
BAZ suppressed ISO-induced collagen synthesis and TGF-β1 secretion in cardiac fibroblasts (CFs); this effect was partially counteracted by IL-6 overexpression. (**A**) Hyp levels in culture supernatants of CFs from each experimental group. (**B**) TGF-β1 concentrations in culture supernatants of CFs from each experimental group. Data are expressed as mean ± SD (*n* = 3 independent experiments). * *p* < 0.05, ** *p* < 0.01, *** *p* < 0.001.

Hyp levels in culture supernatants were measured using a biochemical assay, with results presented in Figure 3A. Compared to the CTL and Veh groups, ISO stimulation led to a marked increase in Hyp concentration in cardiac fibroblast culture supernatants (*p* = 0.00001 or *p* = 0.0004), consistent with a conversion of fibroblasts to a profibrotic phenotype. Pretreatment with 5 μM bazedoxifene (BAZ) significantly attenuated the ISO-induced elevation of Hyp (*p* = 0.0031 vs. ISO model group), indicating that BAZ effectively inhibits ISO-driven collagen deposition. The efficacy of BAZ remained unchanged following transfection with a negative control lentivirus (Lv-NC) (*p* = 0.902). However, the inhibitory effect of BAZ on Hyp generation was considerably reduced by co-transfection with an IL-6 overexpression lentivirus (Lv-IL-6) (*p* = 0.0208 vs. 5 μM BAZ + ISO group), suggesting that IL-6 upregulation partially counteracted the antifibrotic effect of BAZ.

### 3.4. BAZ Inhibits ISO-Induced TGF-β1 Secretion in Cardiac Fibroblasts

ELISA analysis (Figure 3B) demonstrated that ISO stimulation significantly elevated TGF-β1 concentrations in the conditioned medium of cardiac fibroblasts. Pretreatment with 5 μM BAZ markedly reduced TGF-β1 levels (*p* = 0.0026 vs. ISO model group), confirming effective inhibition of the release of this pivotal profibrotic cytokine. There was no statistically significant difference in TGF-β1 levels when comparing the 5 μM BAZ + ISO group to the 5 μM BAZ + Lv-NC + ISO group. Notably, the suppressive effect of BAZ was reversed by exogenous overexpression of IL-6 (*p* = 0.0106 vs. 5 μM BAZ + ISO group), which restored TGF-β1 concentrations to levels that were not statistically different from those observed upon ISO stimulation.

### 3.5. BAZ Inhibits ISO-Induced Migration of Cardiac Fibroblasts

**Figure 4:**
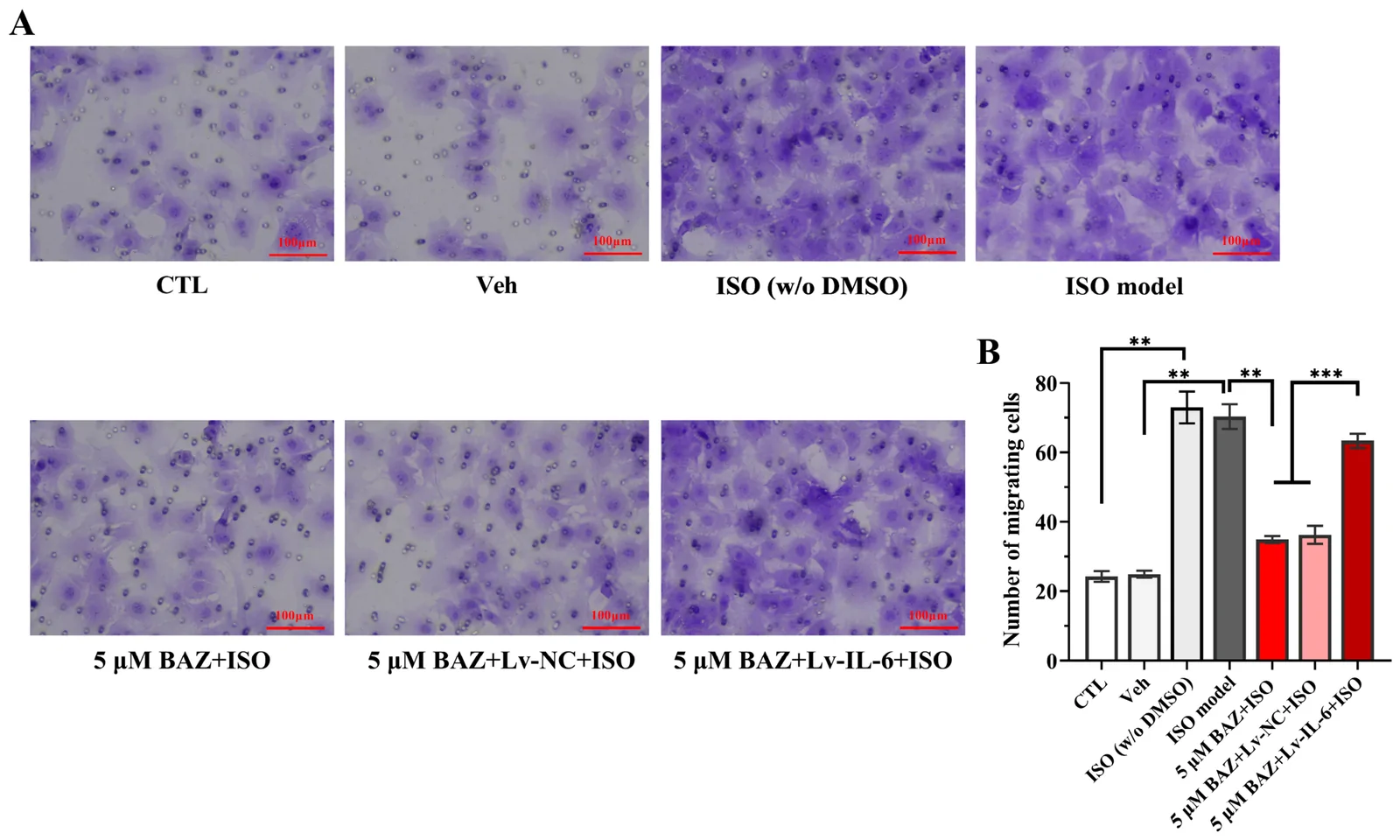
BAZ inhibited ISO-induced cardiac fibroblast migration, an effect that was partially reversed by IL-6 overexpression. (**A**) Representative Transwell migration micrographs. Original magnification: 200×. After crystal violet staining, cells that traversed the microporous membrane and adhered to the lower chamber were stained purple, indicating migratory cells. (**B**) Quantitative analysis of the number of cells that migrated through the membrane for each group. Data are presented as mean ± SD (*n* = 3 independent experiments). ** *p* < 0.01, *** *p* < 0.001.

Transwell migration assays (Figure 4) demonstrated that, relative to the CTL and Veh groups, stimulation with ISO significantly enhanced the transmembrane migratory capacity of cardiac fibroblasts (*p* = 0.0013 or *p* = 0.0011), confirming the successful induction of a pro-migratory fibroblast phenotype by ISO. Pre-treatment with 5 μM BAZ markedly suppressed this ISO-induced migratory activity (*p* = 0.0018 vs. the ISO model group), indicating effective inhibition of pathological fibroblast migration by BAZ. Furthermore, transfection with a negative-control lentivirus (Lv-NC) did not affect BAZ-mediated suppression, ruling out non-specific effects of the viral vector. Compared with the 5 μM BAZ + ISO group, the number of migrated cells in the 5 μM BAZ + Lv-IL-6 + ISO group was significantly restored (*p* = 0.0003), suggesting that IL-6 upregulation partially counteracted the anti-migratory effect of BAZ.

### 3.6. BAZ Suppresses ISO-Induced Cardiac Fibroblast Activation and Collagen Synthesis

**Figure 5:**
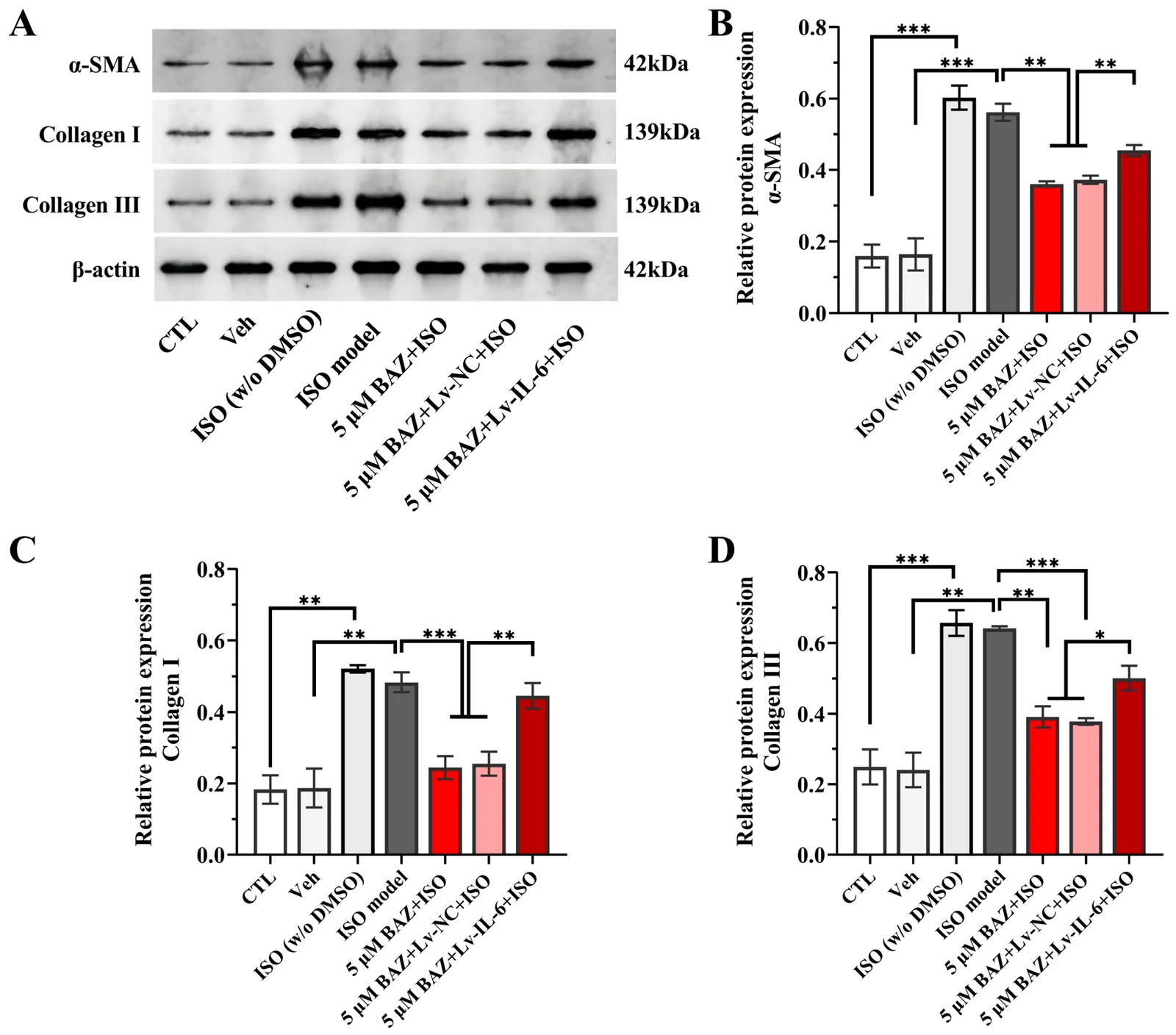
Bazedoxifene inhibits ISO-induced activation of cardiac fibroblasts and collagen synthesis, an effect that is partially reversed by IL-6 overexpression. (**A**) Western blot analysis showing protein expression bands of α-SMA, Collagen I, and Collagen III in cardiac fibroblasts from the respective groups. (**B**–**D**) Quantitative analyses of protein expression levels: α-SMA (**B**), Collagen I (**C**), and Collagen III (**D**). Densitometric values were normalized to β-actin and expressed as relative expression levels. Data are presented as mean ± SD (*n* = 3 independent experiments). * *p* < 0.05, ** *p* < 0.01, *** *p* < 0.001.

To evaluate the effect of BAZ on CF phenotypic conversion and extracellular matrix remodeling, the expression levels of myofibroblast marker α-SMA and fibrotic proteins Collagen I and Collagen III were assessed via Western blot. As illustrated in Figure 5A–D, ISO stimulation led to significant upregulation of α-SMA, Collagen I, and Collagen III protein levels in CFs compared with the CTL and Veh groups (*p* < 0.01 or *p* < 0.001), confirming successful induction of fibroblast-to-myofibroblast transdifferentiation and excessive collagen deposition by ISO. Upon administration of 5 μM BAZ, the protein levels of all three markers were markedly decreased (*p* < 0.01 or *p* < 0.001 vs. the ISO model group), indicating effective inhibition of ISO-driven CF activation and fibrotic progression by BAZ. No change in BAZ efficacy was observed after transfection with negative control virus (Lv-NC), thereby excluding vector-related interference. Further mechanistic rescue experiments revealed that co-transfection with Lv-IL-6 partially restored the protein levels of α-SMA, Collagen I, and Collagen III in CFs compared to the BAZ-treated group (*p* < 0.05 or *p* < 0.01 vs. 5 μM BAZ + ISO group). These results demonstrate that exogenous overexpression of IL-6 (Lv-IL-6) substantially reversed the inhibitory effect of BAZ on fibrotic protein expression.

### 3.7. BAZ Downregulates ISO-Induced IL-6 Expression and Secretion in Cardiac Fibroblasts

**Figure 6:**
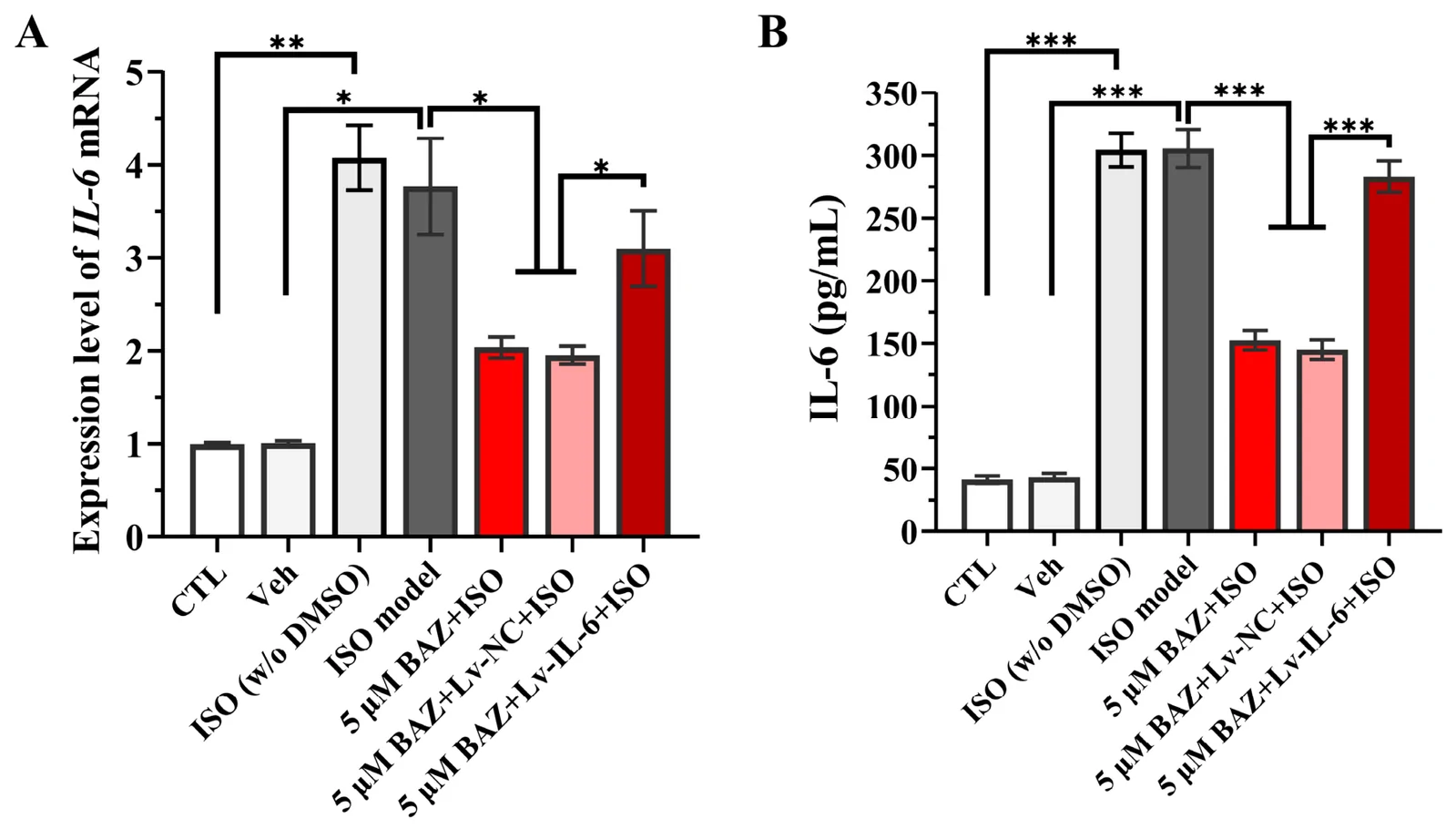
BAZ suppressed ISO-induced IL-6 expression and secretion in cardiac fibroblasts, an effect that was reversed by IL-6 overexpression. (**A**) Relative *IL-6* mRNA expression levels in cardiac fibroblasts from each group were assessed by qRT-PCR. Data are expressed as mean ± SD (*n* = 3 independent experiments). (**B**) IL-6 protein concentrations in cell culture supernatants were quantified by ELISA. Data are expressed as mean ± SD (*n* = 4 independent experiments). * *p* < 0.05, ** *p* < 0.01, *** *p* < 0.001.

To investigate whether BAZ exerts anti-fibrotic effects through modulation of IL-6, both transcriptional and secretory levels of IL-6 were evaluated in primary CFs across experimental groups. As depicted in Figure 6A, qRT-PCR analysis demonstrated that ISO stimulation robustly upregulated *IL-6* mRNA expression in CFs (*p* = 0.0115 vs. vehicle-treated control group), confirming activation of pro-inflammatory and pro-fibrotic signaling pathways. Pre-treatment with 5 μM BAZ significantly attenuated this ISO-induced *IL-6* mRNA elevation (*p* = 0.0246 vs. ISO model group), indicating potent transcriptional suppression of *IL-6* overexpression by BAZ. At the protein level, ELISA measurements of culture supernatants (Figure 6B) mirrored the mRNA findings. ISO elicited a pronounced increase in secreted IL-6 (*p* = 0.00003 vs. veh group), whereas BAZ pre-treatment markedly blunted this response (*p* = 0.00003 vs. ISO model group). Transduction with negative control lentivirus (Lv-NC) did not alter these outcomes, supporting the specificity of BAZ’s pharmacological action. Furthermore, overexpression of IL-6 via lentiviral transduction (Lv-IL-6) significantly reversed the suppressive effects of BAZ on both *IL-6* mRNA and protein secretion, with IL-6 levels restored to levels that showed no statistically significant difference compared with the ISO model group. Collectively, these findings strongly implicate IL-6 as a pivotal mediator of the inflammatory response in CFs and underscore its important role in mediating the anti-migratory and anti-inflammatory actions of BAZ, likely through modulation of the IL-6/STAT3 signaling axis.

### 3.8. BAZ Inhibits ISO-Induced Activation of the IL-6/STAT3 Signaling Pathway

**Figure 7:**
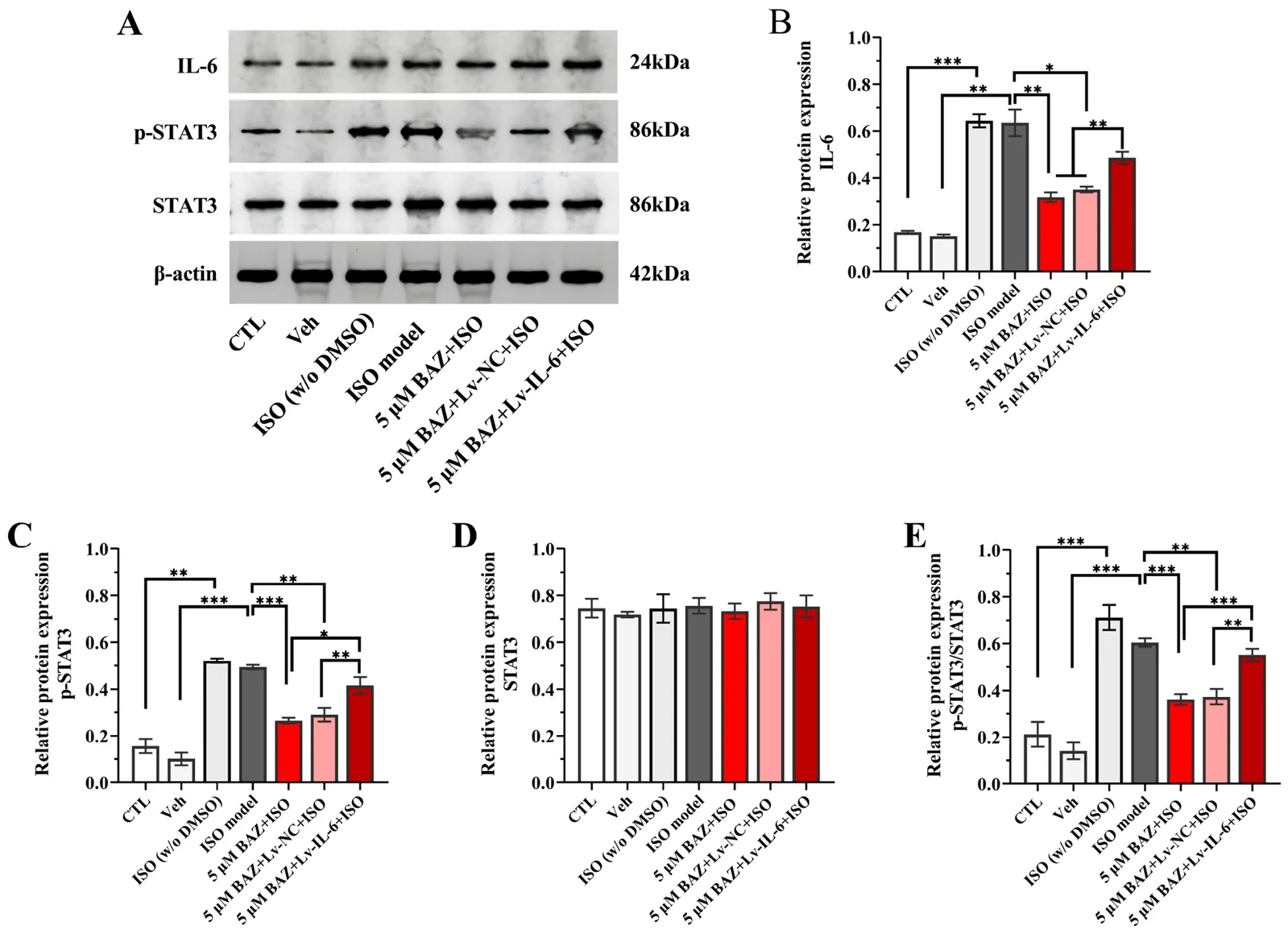
BAZ suppresses IL-6 expression, thereby inhibiting ISO-induced STAT3 phosphorylation in cardiac fibroblasts. (**A**) Representative Western blot images showing the protein expression levels of IL-6, phosphorylated STAT3 (p-STAT3), and total STAT3 in cardiac fibroblasts across all experimental groups. (**B**–**D**) Quantitative analyses of IL-6 (**B**), p-STAT3 (**C**), and total STAT3 (**D**) expression, with grayscale intensities normalized to β-actin and presented as relative expression values. (**E**) Ratio of p-STAT3 to total STAT3, indicating the activation state of STAT3 phosphorylation. Data are expressed as mean ± SD (*n* = 3 independent experiments). * *p* < 0.05, ** *p* < 0.01, *** *p* < 0.001.

To investigate the precise mechanism through which BAZ modulates IL-6 downstream signaling, Western blot analysis was performed to assess the protein expression levels of IL-6 and the phosphorylation status of its canonical downstream effector, STAT3, in cardiac fibroblasts from each experimental group. As depicted in Figure 7A–E, ISO stimulation significantly elevated intracellular IL-6 protein expression (*p* = 0.0041 vs. Veh group) and induced the specific phosphorylation of STAT3 (p-STAT3), reflected by a marked increase in the p-STAT3/STAT3 ratio (*p* = 0.0003 vs. Veh group), while total STAT3 protein levels remained unchanged across all groups. Following treatment with 5 μM BAZ, not only was IL-6 protein expression substantially reduced, but p-STAT3 expression was also significantly suppressed (*p* = 0.00002 vs. ISO model group), indicating that BAZ effectively inhibits the ISO-activated IL-6/STAT3 signaling cascade.

Further mechanistic validation demonstrated that exogenous overexpression of IL-6 via lentiviral transduction (Lv-IL-6) partially reversed the inhibitory effect of BAZ on this signaling pathway. Specifically, co-treatment with Lv-IL-6 in the presence of BAZ restored intracellular IL-6 protein levels (*p* = 0.0011 vs. 5 μM BAZ + ISO group), accompanied by a significant recovery of p-STAT3 expression and the p-STAT3/STAT3 ratio (*p* = 0.0101 or *p* = 0.0008 vs. 5 μM BAZ + ISO group). This rescue experiment further suggests that IL-6 contributes to BAZ-mediated suppression of STAT3 phosphorylation and the attenuation of fibrotic responses, indicating IL-6 as a functionally important mediator in the anti-fibrotic action of BAZ.

## 4. Discussion

In the present study, we demonstrate that BAZ effectively inhibits ISO-induced activation, proliferation, migration, and collagen deposition in neonatal rat cardiac fibroblasts in vitro. Mechanistically, BAZ downregulates IL-6 expression and secretion in cardiac fibroblasts, thereby blocking the aberrant activation of IL-6/STAT3 signaling. Lentiviral-mediated IL-6 overexpression reconstitution experiments further confirmed the central mediatory role of IL-6 in the antifibrotic effect of BAZ. These findings extend the pharmacological profile of BAZ from oncology and osteoporosis to myocardial fibrosis, providing new experimental evidence supporting drug repurposing.

Cardiac fibroblasts serve as key effector cells in the initiation and progression of myocardial fibrosis [4]. Upon pathological stimulation, quiescent cardiac fibroblasts become activated into contractile myofibroblasts, leading to excessive secretion of extracellular matrix (ECM) and subsequent myocardial interstitial fibrosis [2]. BAZ was observed to effectively suppress the abnormal proliferation of neonatal rat cardiac fibroblasts induced by ISO. Flow cytometry analysis demonstrated that BAZ treatment significantly increased the proportion of cells in the G_0_/G_1_ phase while markedly decreasing the proportion in the S phase, indicating an antiproliferative effect mediated by blocking the transition of cardiac fibroblasts from the G_0_/G_1_ to the S phase. These findings align with previously reported antiproliferative activities of BAZ in other cell types. Within the bone microenvironment, BAZ acts as an estrogen receptor agonist, inhibiting osteoclast activity and preserving bone density, thereby indirectly suppressing the proliferation of bone-resorptive cells [19,20]. In endometrial and mammary tissues, BAZ exhibits clear estrogen receptor antagonism, dose-dependently inhibiting estrogen-induced epithelial cell proliferation [21]. During B lymphocyte development, BAZ mildly reduced the numbers of late-stage bone marrow pre-B cells and splenic transitional 1 (T1) B cells, without significantly interfering with the proliferation of early B-lymphoid progenitors [15,22]. Furthermore, Transwell assays showed that BAZ significantly inhibited ISO-induced migration of cardiac fibroblasts. Fibroblast migration is a critical step in the formation and dissemination of fibrotic lesions; BAZ-mediated blockade of this process further supports its antifibrotic potential. Combined application of BAZ and paclitaxel was reported to suppress breast cancer cell viability, migration, and tumor growth, and to induce apoptosis [23]. Regarding collagen deposition, Western blot analysis revealed that BAZ markedly downregulated the protein expression of α-SMA, Collagen I, and Collagen III, while ELISA indicated a reduction in hydroxyproline content in culture supernatants. These results are highly consistent with observations by Shi et al. in a transverse aortic constriction mouse model, which confirmed that BAZ significantly downregulated the transcriptional levels of fibrotic genes Col1A1, Col3A1, and MMP2 in myocardial tissue [16]. Collectively, these findings further support the potential application of BAZ as an anti-myocardial fibrosis agent.

Notably, the viability of targeting IL-6 signaling as an anti-myocardial fibrosis approach has been further substantiated by recent investigations. In a cardiac fibrosis model induced by radiotherapy combined with immunotherapy, the IL-6 receptor inhibitor tocilizumab effectively alleviated acute cardiac injury, inflammation, and fibrosis through suppression of IL-6-mediated fibroblast–macrophage interactions [24]. In the context of high-fat diet–induced myocardial fibrosis, administration of an IL-6 neutralizing antibody completely reversed elevations in angiotensin II and IL-6, thereby inhibiting collagen synthesis [25]. In heart failure models, the class I HDAC inhibitor mocetinostat reduced IL-6 levels and p-STAT3 expression, resulting in decreased scar area and collagen deposition [26]. Diosgenin reversed arsenic-induced endothelial-to-mesenchymal transition via modulation of the glucocorticoid receptor/IL-6 axis [27]. Alpinetin suppressed the TLR4/MyD88/NF-κB signaling pathway, downregulated inflammatory mediators including IL-6, and attenuated inflammation and fibrosis in the infarct zone [28]. Collectively, these lines of evidence indicate that targeting the IL-6/STAT3 axis represents a promising anti-myocardial fibrosis strategy, and BAZ, as an approved drug, confers unique advantages for clinical translation.

Under ISO stimulation, cardiac fibroblasts (CFs), rather than cardiomyocytes, were identified as the primary cellular source of IL-6 [29]. In this study, ISO induced a significant upregulation of IL-6 mRNA and protein levels in CFs, along with phosphorylation of STAT3 at Tyr705, consistent with previous reports. Upon IL-6 binding to its receptor, JAK2 was activated and STAT3 was phosphorylated at Tyr705, promoting dimerization and nuclear translocation, thereby initiating downstream gene transcription [30,31]. Following BAZ treatment, IL-6 expression and STAT3 phosphorylation were markedly suppressed. Mechanistically, BAZ was found to directly bind to the D1 domain of gp130, thereby blocking the IL-6-gp130 interaction and preventing intracellular signal propagation [14,15]. Validation has been obtained across multiple malignancies—including pancreatic cancer, hepatocellular carcinoma [14], triple-negative breast cancer [32], ovarian cancer [33], osteosarcoma, colorectal cancer [34], gastric cancer [35], and rhabdomyosarcoma [36]—where BAZ exerted antitumor activity via inhibition of the IL-6/gp130/STAT3 pathway. Similarly, in models of atherosclerosis [37], abdominal aortic aneurysm [38], pressure-overload cardiac remodeling [16], septic cardiomyopathy [39], and autoimmune myocarditis [40], BAZ attenuated inflammation and tissue injury through suppression of IL-6/STAT3 signaling. Collectively, these findings indicate broad anti-inflammatory and antitumor activities, as well as protective effects in cardiovascular and immune-mediated diseases. The present study extended this mechanism to CFs and, for the first time, employed IL-6 overexpression via lentiviral rescue to demonstrate that exogenous IL-6 overexpression partially reversed BAZ-induced cell cycle arrest, migration suppression, collagen synthesis attenuation, and inhibition of STAT3 phosphorylation. We therefore propose that BAZ may exert dual actions on the IL-6/STAT3 axis: direct antagonism of gp130-mediated STAT3 activation, and indirect suppression of IL-6 expression, which together disrupt a positive feedback loop that sustains the fibrotic phenotype. Future studies employing acute time-course analyses and complementary approaches (e.g., recombinant IL-6 supplementation, IL-6 neutralization, or constitutively active STAT3 constructs) would be valuable to further dissect the hierarchical relationship between these mechanisms.

**Figure 8:**
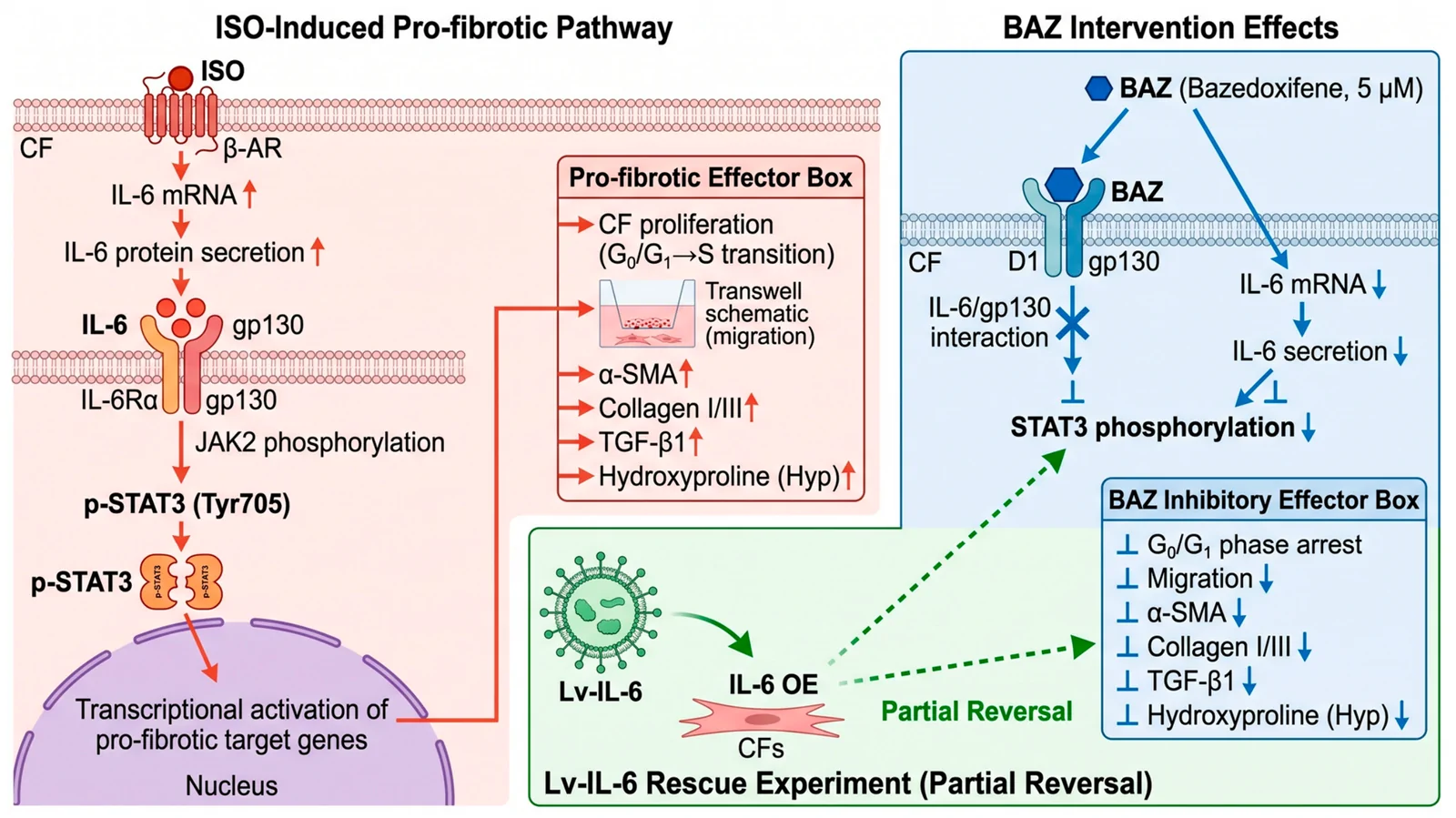
Schematic illustration of the proposed mechanism by which bazedoxifene attenuates ISO-induced cardiac fibroblast activation and fibrotic phenotype. In this figure, arrows indicate directionality of signaling and regulatory effects: solid arrows represent direct activation or promotion, whereas dashed arrows denote indirect or downstream effects; red arrows indicate upregulation or increased activity, and blue or green arrows indicate inhibitory effects (as annotated). Under ISO stimulation (left), cardiac fibroblasts upregulate IL-6 expression and secretion, which binds to IL-6Rα and the co-receptor gp130, activating downstream JAK2/STAT3 signaling. Phosphorylated STAT3 (p-STAT3, Tyr705) dimerizes and translocates to the nucleus, where it drives the transcription of pro-fibrotic target genes, leading to CF proliferation (G_0_/G_1_ to S phase transition), migration, myofibroblast transdifferentiation (α-SMA upregulation), and excessive collagen synthesis (Collagen I/III and hydroxyproline). Concurrently, IL-6/STAT3 signaling enhances TGF-β1 production, while TGF-β1 further amplifies IL-6 expression, establishing a positive feedback loop that sustains the fibrotic phenotype. BAZ pretreatment (right) exerts dual actions on the IL-6/STAT3 axis: (1) direct antagonism of gp130-mediated STAT3 activation by binding to the D1 domain of gp130, and (2) indirect suppression of IL-6 expression at both the transcriptional and protein levels, thereby reducing ligand availability and disrupting the IL-6/STAT3/TGF-β1 feedforward loop. These combined effects result in G_0_/G_1_ cell cycle arrest, inhibition of migration, suppression of myofibroblast activation, and reduced collagen synthesis. Lentiviral-mediated IL-6 overexpression (Lv-IL-6, bottom) partially reverses the anti-fibrotic effects of BAZ, confirming that IL-6 serves as a functionally important mediator of BAZ’s pharmacological action. Abbreviations: BAZ, bazedoxifene; CFs, cardiac fibroblasts; ECM, extracellular matrix; gp130, glycoprotein 130; Hyp, hydroxyproline; IL-6, interleukin-6; ISO, isoproterenol; JAK2, Janus kinase 2; Lv-IL-6, IL-6-overexpressing lentivirus; OE, overexpression; p-STAT3, phosphorylated STAT3; STAT3, signal transducer and activator of transcription 3; TGF-β1, transforming growth factor-beta 1; α-SMA, alpha-smooth muscle actin.

The secretion of TGF-β1 induced by ISO was attenuated by BAZ, and this inhibitory effect was partially reversed upon overexpression of IL-6. These findings suggest a cross-regulatory interaction between the IL-6/STAT3 and TGF-β1 signaling pathways. In diverse physiological and pathological settings, these two pathways converge into a highly integrated and mutually regulatory network. For instance, in pancreatic stellate cells, IL-6 promotes TGF-β1 production through STAT3 signaling [41]. In intestinal fibrosis associated with Crohn’s disease, IL-6 activation correlates with a noncanonical pattern of STAT3 phosphorylation—characterized by elevated p-STAT3 at Ser727 and reduced p-STAT3 at Tyr705—leading to increased TGF-β1 expression and subsequent collagen deposition [42]. In pulmonary fibrosis, TGF-β1 induces fibroblast activation, whereas IL-6 blockade reverses TGF-β1-driven fibroblast-to-myofibroblast transition [43]. Conversely, TGF-β1 upregulates IL-6 expression, thereby activating the IL-6/STAT3 pathway. In murine models of cirrhosis or hepatocellular carcinoma, a positive correlation between TGF-β1 and IL-6 levels has been observed [44]. In cholangiocytes, prolonged exposure to TGF-β1 markedly enhances IL-6 secretion and receptor expression, subsequently activating downstream IL-6/STAT3 proliferative signaling [45]. In renal fibroblasts, TGF-β1 directly induces STAT3 phosphorylation, albeit to a lesser extent than IL-6; this mechanism is considered to contribute synergistically to fibrosis progression [46]. Targeted inhibition of JAK2/STAT3 or blockade of IL-6 has been shown to attenuate TGF-β1-induced myofibroblast activation, collagen deposition, and inflammatory responses, thereby alleviating pulmonary fibrosis [43]. BAZ may exert its antifibrotic effects by disrupting a positive feedback loop between the IL-6/STAT3 and TGF-β1 pathways, providing a theoretical foundation for clinical translation. The proposed molecular mechanism of BAZ in attenuating ISO-induced cardiac fibroblast activation and fibrotic phenotype is summarized schematically in Figure 8.

Bazedoxifene (BAZ) is approved in the United States in fixed combination with conjugated estrogens (0.45 mg CE + 20 mg BAZ, marketed as DUAVEE^®^) for the treatment of moderate-to-severe vasomotor symptoms associated with menopause and for the prevention of postmenopausal osteoporosis in women with a uterus. This tissue-selective estrogen complex (TSEC) pairs a SERM with estrogen, providing endometrial and breast protection alongside estrogenic benefits on bone and vasomotor symptoms [14]. Nonetheless, the cardiovascular profile of TSEC warrants further investigation, and Mehta et al. have emphasized the importance of individualized risk assessment and shared decision-making in its clinical use [47]. In recent years, the cardiovascular protective properties of BAZ have garnered increasing recognition. Existing studies have demonstrated its beneficial effects on cardiovascular tissues [16,37,38,39,40], without any evidence of breast tissue proliferation or an elevated risk of breast cancer [48,49,50]. From a clinical translation perspective, BAZ offers several advantages as a repurposed drug: (1) its safety profile is well-established from extensive clinical use in osteoporosis, with known pharmacokinetics, dosing regimens, and adverse effect profiles; (2) its oral bioavailability and favorable tissue distribution facilitate convenient administration; and (3) the availability of generic formulations may reduce treatment costs. However, several challenges must be addressed before clinical application in myocardial fibrosis: (a) the optimal dosing regimen for cardiovascular indications may differ from that used in osteoporosis and requires dedicated dose-finding studies; (b) the long-term cardiovascular safety of BAZ, particularly in male patients and in combination with standard heart failure therapies, needs to be established; (c) the potential estrogenic effects on other tissues (e.g., endometrium, breast) should be carefully monitored, although BAZ has demonstrated a favorable tissue-selective profile; and (d) patient selection criteria—including sex, menopausal status, and concomitant medications—will need to be defined to maximize therapeutic benefit while minimizing risks. This study presents novel experimental evidence supporting the repurposing of BAZ, suggesting that it may serve as a promising candidate for the treatment of myocardial fibrosis, and provides a mechanistic foundation for future clinical investigation.

Several limitations of this study should be acknowledged. First, all experiments were conducted using cultured neonatal rat cardiac fibroblasts ex vivo. Neonatal cardiac fibroblasts exhibit fundamental differences from their adult counterparts, including a higher basal proliferative rate, a distinct profile of extracellular matrix production, and divergent responses to pathological stimuli. Furthermore, species-specific disparities between rat models and human cardiac fibroblasts—reflected in differences in developmental stage, cellular heterogeneity, and the complexity of the disease microenvironment—may constrain the translational relevance of findings derived from animal studies [51,52]. Although neonatal cardiac fibroblast models have long served as a cornerstone for mechanistic investigations owing to their robust proliferative capacity and experimental accessibility, confirmation in adult cardiac fibroblasts, and ultimately in human cardiac fibroblasts or induced pluripotent stem cell–derived fibroblasts, is essential to establish the clinical relevance of BAZ-mediated antifibrotic effects. This limitation has been explicitly acknowledged and designated as a priority for future investigation. Second, although the ISO-induced cardiac fibroblast activation model is well established and reliable, it does not fully recapitulate the complex neurohumoral microenvironment present in vivo. Third, while IL-6 overexpression rescue experiments confirmed a central role for IL-6 in the pharmacological mechanism of BAZ, whether BAZ mediates its antifibrotic effects through additional signaling pathways (e.g., NF-κB, MAPK) remains to be explored. Furthermore, the effects of BAZ on other members of the IL-6 family (e.g., IL-11) were not validated in CFs.

In summary, BAZ suppressed ISO-induced IL-6 expression and secretion in CFs, thereby attenuating aberrant activation of the IL-6/STAT3 signaling pathway and effectively inhibiting fibroblast proliferation, migration, activation, and collagen deposition. IL-6 overexpression rescue experiments confirmed that IL-6 acts as an important mediator of the antifibrotic effects elicited by BAZ. These findings reveal the potential utility of BAZ as an inhibitor of the IL-6/STAT3 pathway for attenuation of cardiac fibrosis, provide in vitro evidence supporting the involvement of IL-6/STAT3 signaling in its anti-fibrotic mechanism. Nevertheless, the translational relevance of these findings requires validation in in vivo models and, ultimately, in clinical studies.

## Data Availability

The original contributions presented in this study are included in the article. Further inquiries can be directed to the corresponding author.

## Abbreviations

The following abbreviations are used in this manuscript:

BAZ	Bazedoxifene
CFs	Cardiac fibroblasts
ECM	Extracellular matrix
MI	myocardial infarction
Hyp	Hydroxyproline
IL-6	Interleukin-6
ISO	Isoproterenol
Lv-IL-6	IL-6-overexpressing lentivirus
Lv-NC	Negative control lentivirus
PRO	Propranolol
SERM	Selective estrogen receptor modulator
STAT3	Signal transducer and activator of transcription 3
TGF-β1	Transforming growth factor-beta 1
α-SMA	Alpha-smooth muscle actin
Veh	Vehicle
CTL	Control
TBST	Tris-buffered saline with Tween 20
SDS-PAGE	Sodium dodecyl sulfate-polyacrylamide gel electrophoresis
RIPA	Radioimmunoprecipitation assay
qRT-PCR	Quantitative real-time polymerase chain reaction
PVDF	Polyvinylidene difluoride
PI	Propidium iodide
PBS	Phosphate-buffered saline
MOI	Multiplicity of infection
FBS	Fetal bovine serum
ELISA	Enzyme-linked immunosorbent assay
EDTA	Ethylenediaminetetraacetic acid
DMSO	Dimethyl sulfoxide
DAPI	4′,6-Diamidino-2-phenylindole
CCK-8	Cell Counting Kit-8
BSA	Bovine serum albumin
gp130	Glycoprotein 130
HDAC	Histone deacetylase
IL-11	Interleukin-11
JAK2	Janus kinase 2
MAPK	Mitogen-activated protein kinase
NF-κB	Nuclear factor kappa-light-chain-enhancer of activated B cells

## References

[1] Nauffal V., Di Achille P., Klarqvist M.D.R., Cunningham J.W., Hill M.C., Pirruccello J.P., Weng L.C., Morrill V.N., Choi S.H., Khurshid S. (2023). Genetics of myocardial interstitial fibrosis in the human heart and association with disease. Nat. Genet..

[2] Pereira-Sousa D., Guillamat P., Niro F., Vinarský V., Fernandes S., Cassani M., Pagliari S., Trepat X., Rasponi M., Oliver-De La Cruz J. (2026). Cardiac fibroblast anisotropy is determined by YAP-dependent cellular contractility and ECM production. Biomaterials.

[3] Nagy D., Radovits T., Bálint T., Horváth Z., Kocsis-Balogh P., Tóth Á.G., Oláh A., Sayour A.A., Barta B.A., Merkely B. (2026). The anti-fibrotic effects of novel heart failure pharmacotherapies in advanced heart failure patients. Br. J. Pharmacol..

[4] Van Linthout S., Matz I., González A., Davis J. (2026). Cardiac fibroblasts in myocardial injury and heart failure. Eur. Heart J..

[5] Kumar S., Wang G., Zheng N., Cheng W., Ouyang K., Lin H., Liao Y., Liu J. (2019). HIMF (Hypoxia-Induced Mitogenic Factor)-IL (Interleukin)-6 Signaling Mediates Cardiomyocyte-Fibroblast Crosstalk to Promote Cardiac Hypertrophy and Fibrosis. Hypertension.

[6] Müller J., Gorressen S., Grandoch M., Feldmann K., Kretschmer I., Lehr S., Ding Z., Schmitt J.P., Schrader J., Garbers C. (2014). Interleukin-6-dependent phenotypic modulation of cardiac fibroblasts after acute myocardial infarction. Basic Res. Cardiol..

[7] Li H., Bian Y. (2024). Fibroblast-derived interleukin-6 exacerbates adverse cardiac remodeling after myocardial infarction. Korean J. Physiol. Pharmacol..

[8] Wu X., Cao Y., Xiao H., Feng J., Lin J. (2024). Bazedoxifene Suppresses the Growth of Osteosarcoma Cells by Inhibiting IL-6 and IL-11/GP130 Signaling Pathway. J. Pediatr. Hematol. Oncol..

[9] Chakraborty D., Šumová B., Mallano T., Chen C.W., Distler A., Bergmann C., Ludolph I., Horch R.E., Gelse K., Ramming A. (2017). Activation of STAT3 integrates common profibrotic pathways to promote fibroblast activation and tissue fibrosis. Nat. Commun..

[10] Liu Y., Wang J., Cai H., Wang Z., Lu R., Liu X., Wang M., Wang W., Guo J., Ma G. (2026). Neutrophil methylmalonic acid promotes microthrombus formation and adverse cardiac remodeling post-myocardial infarction through activating IL-6 signaling pathway-mediated NETosis. BMC Med..

[11] Men L., Guo J., Cao Y., Huang B., Wang Q., Huo S., Wang M., Peng D., Peng L., Shi W. (2023). IL-6/gp130/STAT3 signaling contributed to the activation of the PERK arm of the unfolded protein response in response to chronic β-adrenergic stimulation. Free Radic. Biol. Med..

[12] Cong Z., Ivetic A., Parsons M. (2026). Monocytes and neutrophils promote cardiac fibroblast pro-fibrotic phenotypes through IL-6 and MIF. Front. Cell Dev. Biol..

[13] Wang J.H., Zhao L., Pan X., Chen N.N., Chen J., Gong Q.L., Su F., Yan J., Zhang Y., Zhang S.H. (2016). Hypoxia-stimulated cardiac fibroblast production of IL-6 promotes myocardial fibrosis via the TGF-β1 signaling pathway. Lab. Investig..

[14] Tella S.H., Gallagher J.C. (2013). Bazedoxifene + conjugated estrogens in HT for the prevention of osteoporosis and treatment of vasomotor symptoms associated with the menopause. Expert Opin. Pharmacother..

[15] Ma H., Yan D., Wang Y., Shi W., Liu T., Zhao C., Huo S., Duan J., Tao J., Zhai M. (2019). Bazedoxifene exhibits growth suppressive activity by targeting interleukin-6/glycoprotein 130/signal transducer and activator of transcription 3 signaling in hepatocellular carcinoma. Cancer Sci..

[16] Shi W., Ma H., Liu T., Yan D., Luo P., Zhai M., Tao J., Huo S., Guo J., Li C. (2020). Inhibition of Interleukin-6/glycoprotein 130 signalling by Bazedoxifene ameliorates cardiac remodelling in pressure overload mice. J. Cell. Mol. Med..

[17] Sun M., Yu H., Zhang Y., Li Z., Gao W. (2015). MicroRNA-214 Mediates Isoproterenol-induced Proliferation and Collagen Synthesis in Cardiac Fibroblasts. Sci. Rep..

[18] Lu Y., Li Y., Zhu C., Guo M., Liu X., Chen X. (2025). *Cinnamomum migao* H.W. Li Ethanol-Water Extract Suppresses IL-6 Production in Cardiac Fibroblasts: Mechanisms Elucidated via UPLC-Q-TOF-MS, Network Pharmacology, and Experimental Assays. Curr. Issues Mol. Biol..

[19] Lewiecki E.M. (2007). Bazedoxifene and bazedoxifene combined with conjugated estrogens for the management of postmenopausal osteoporosis. Expert Opin. Investig. Drugs.

[20] Lello S., Capozzi A., Scambia G. (2017). The Tissue-Selective Estrogen Complex (Bazedoxifene/Conjugated Estrogens) for the Treatment of Menopause. Int. J. Endocrinol..

[21] Pickar J.H., Komm B.S. (2015). Selective estrogen receptor modulators and the combination therapy conjugated estrogens/bazedoxifene: A review of effects on the breast. Post Reprod. Health.

[22] Bernardi A.I., Andersson A., Grahnemo L., Nurkkala-Karlsson M., Ohlsson C., Carlsten H., Islander U. (2014). Effects of lasofoxifene and bazedoxifene on B cell development and function. Immun. Inflamm. Dis..

[23] Fu S., Chen X., Lo H.W., Lin J. (2019). Combined bazedoxifene and paclitaxel treatments inhibit cell viability, cell migration, colony formation, and tumor growth and induce apoptosis in breast cancer. Cancer Lett..

[24] Luo Y., Yu Y., Zeng Z., Chen Y., Yi Y., Lu Z., Zeng F., Xu P., Luo D., Chen L. (2026). Decoding the Cardiac Immune Microenvironment and Fibroblast Crosstalk in Radiotherapy Combined with Immunotherapy-Induced Cardiac Fibrosis Based on Single-Cell Transcriptomic Analysis. Adv. Sci..

[25] Eid R.A., Alkhateeb M.A., El-Kott A.F., Eleawa S.M., Zaki M.S.A., Alaboodi S.A., Salem Al-Shudiefat A.A., Aldera H., Alnamar N.M., Alassiri M. (2019). A high-fat diet rich in corn oil induces cardiac fibrosis in rats by activating JAK2/STAT3 and subsequent activation of ANG II/TGF-1β/Smad3 pathway: The role of ROS and IL-6 trans-signaling. J. Food Biochem..

[26] Nural-Guvener H., Zakharova L., Feehery L., Sljukic S., Gaballa M. (2015). Anti-Fibrotic Effects of Class I HDAC Inhibitor, Mocetinostat Is Associated with IL-6/Stat3 Signaling in Ischemic Heart Failure. Int. J. Mol. Sci..

[27] Cui H., Xu W., Liu L., Hong Y., Lou H., Tang P., Lin Y., Xu H., Xie M., Du M. (2024). Diosgenin alleviates arsenic trioxide induced cardiac fibrosis by inhibiting endothelial mesenchymal transition. Phytomedicine.

[28] Feng M., Chen X., Huang F., Chen L., Liu C., Li W., Li Y., Chen S., Deng Z., Wei Z. (2025). Alpinetin Alleviates Cardiac Inflammation and Remodeling via TLR4/MyD88/NF-κB Signaling Pathway in Rats with Acute Myocardial Infarction. Int. J. Mol. Sci..

[29] Yin F., Li P., Zheng M., Chen L., Xu Q., Chen K., Wang Y.Y., Zhang Y.Y., Han C. (2003). Interleukin-6 family of cytokines mediates isoproterenol-induced delayed STAT3 activation in mouse heart. J. Biol. Chem..

[30] Taher M.Y., Davies D.M., Maher J. (2018). The role of the interleukin (IL)-6/IL-6 receptor axis in cancer. Biochem. Soc. Trans..

[31] Wang Y., van Boxel-Dezaire A.H., Cheon H., Yang J., Stark G.R. (2013). STAT3 activation in response to IL-6 is prolonged by the binding of IL-6 receptor to EGF receptor. Proc. Natl. Acad. Sci. USA.

[32] Tian J., Chen X., Fu S., Zhang R., Pan L., Cao Y., Wu X., Xiao H., Lin H.J., Lo H.W. (2019). Bazedoxifene is a novel IL-6/GP130 inhibitor for treating triple-negative breast cancer. Breast Cancer Res. Treat..

[33] Park S.A., Kim L.K., Park H.M., Kim H.J., Heo T.H. (2022). Inhibition of GP130/STAT3 and EMT by combined bazedoxifene and paclitaxel treatment in ovarian cancer. Oncol. Rep..

[34] Dmello R.S., Palmieri M., Thilakasiri P.S., Doughty L., Nero T.L., Poh A.R., To S.Q., Lee E.F., Douglas Fairlie W., Mielke L. (2024). Combination of bazedoxifene with chemotherapy and SMAC-mimetics for the treatment of colorectal cancer. Cell Death Dis..

[35] Thilakasiri P., Huynh J., Poh A.R., Tan C.W., Nero T.L., Tran K., Parslow A.C., Afshar-Sterle S., Baloyan D., Hannan N.J. (2019). Repurposing the selective estrogen receptor modulator bazedoxifene to suppress gastrointestinal cancer growth. EMBO Mol. Med..

[36] Xiao H., Bid H.K., Chen X., Wu X., Wei J., Bian Y., Zhao C., Li H., Li C., Lin J. (2017). Repositioning Bazedoxifene as a novel IL-6/GP130 signaling antagonist for human rhabdomyosarcoma therapy. PLoS ONE.

[37] Luo P., Wang Y., Zhao C., Guo J., Shi W., Ma H., Liu T., Yan D., Huo S., Wang M. (2021). Bazedoxifene exhibits anti-inflammation and anti-atherosclerotic effects via inhibition of IL-6/IL-6R/STAT3 signaling. Eur. J. Pharmacol..

[38] Yan D., Ma H., Shi W., Luo P., Liu T., Guo J., Zhai M., Tao J., Huo S., Li C. (2020). Bazedoxifene Attenuates Abdominal Aortic Aneurysm Formation via Downregulation of Interleukin-6/Glycoprotein 130/Signal Transducer and Activator of Transcription 3 Signaling Pathway in Apolipoprotein E-Knockout Mice. Front. Pharmacol..

[39] Jiang T., Peng D., Shi W., Guo J., Huo S., Men L., Zhang C., Li S., Lv J., Lin L. (2022). IL-6/STAT3 Signaling Promotes Cardiac Dysfunction by Upregulating FUNDC1-Dependent Mitochondria-Associated Endoplasmic Reticulum Membranes Formation in Sepsis Mice. Front. Cardiovasc. Med..

[40] Wang J., Liu T., Chen X., Jin Q., Chen Y., Zhang L., Han Z., Chen D., Li Y., Lv Q. (2021). Bazedoxifene Regulates Th17 Immune Response to Ameliorate Experimental Autoimmune myocarditis via Inhibition of STAT3 Activation. Front. Pharmacol..

[41] Zheng M., Li H., Sun L., Brigstock D.R., Gao R. (2021). Interleukin-6 participates in human pancreatic stellate cell activation and collagen I production via TGF-β1/Smad pathway. Cytokine.

[42] Li C., Iness A., Yoon J., Grider J.R., Murthy K.S., Kellum J.M., Kuemmerle J.F. (2015). Noncanonical STAT3 activation regulates excess TGF-β1 and collagen I expression in muscle of stricturing Crohn’s disease. J. Immunol..

[43] Hu X., Tan J., Wang Y., Luan R., Ding D., Yue M., Zhao M., Xue Q., Yang J. (2025). Blocking IL-6 down-regulates PD-L1 expression and mitigates pulmonary fibrosis by inhibiting the STAT3 signaling pathway. Cell Signal..

[44] Hu N., Yan N., Zhao T., Hu L. (2026). Effects of curcumin on the signal transducer and activator of transcription 3 signaling pathway and bile acid enterohepatic circulation in liver cancer mice model. Pak. J. Pharm. Sci..

[45] Yi J., Jeon B.Y., Jeong J.H., Pak J.H. (2025). Transforming growth factor-β1 triggers the proliferation of human cholangiocyte spheroids via interleukin-6-mediated STAT3 signaling. Mol. Biol. Rep..

[46] Chen W., Yuan H., Cao W., Wang T., Chen W., Yu H., Fu Y., Jiang B., Zhou H., Guo H. (2019). Blocking interleukin-6 trans-signaling protects against renal fibrosis by suppressing STAT3 activation. Theranostics.

[47] Mehta J., Kling J.M., Manson J.E. (2021). Risks, Benefits, and Treatment Modalities of Menopausal Hormone Therapy: Current Concepts. Front. Endocrinol..

[48] Mitwally M.F. (2008). Bazedoxifene: A selective estrogen-receptor modulator. Womens Health.

[49] Genant H.K. (2011). Bazedoxifene: A new selective estrogen receptor modulator for postmenopausal osteoporosis. Menopause Int..

[50] Archer D.F., Pinkerton J.V., Utian W.H., Menegoci J.C., de Villiers T.J., Yuen C.K., Levine A.B., Chines A.A., Constantine G.D. (2009). Bazedoxifene, a selective estrogen receptor modulator: Effects on the endometrium, ovaries, and breast from a randomized controlled trial in osteoporotic postmenopausal women. Menopause.

[51] Tani H., Sadahiro T., Yamada Y., Isomi M., Yamakawa H., Fujita R., Abe Y., Akiyama T., Nakano K., Kuze Y. (2023). Direct Reprogramming Improves Cardiac Function and Reverses Fibrosis in Chronic Myocardial Infarction. Circulation.

[52] Jonsson M.K.B., Hartman R.J.G., Ackers-Johnson M., Tan W.L.W., Lim B., van Veen T.A.B., Foo R.S. (2016). A Transcriptomic and Epigenomic Comparison of Fetal and Adult Human Cardiac Fibroblasts Reveals Novel Key Transcription Factors in Adult Cardiac Fibroblasts. JACC Basic Transl. Sci..

